# Assessing Breast Cancer Survivors’ Perceptions of Using Voice-Activated Technology to Address Insomnia: Feasibility Study Featuring Focus Groups and In-Depth Interviews

**DOI:** 10.2196/15859

**Published:** 2020-05-26

**Authors:** Hannah Arem, Remle Scott, Daniel Greenberg, Rebecca Kaltman, Daniel Lieberman, Daniel Lewin

**Affiliations:** 1 Department of Epidemiology Milken Institute School of Public Health George Washington University Washington, DC United States; 2 George Washington Cancer Center Washington, DC United States; 3 MediaRez Washington, DC United States; 4 Division of Hematology and Oncology Medical Faculty Associates George Washington University Washington, DC United States; 5 Department of Psychiatry and Behavioral Sciences Medical Faculty Associates George Washington University Washington, DC United States; 6 Children's National Health System Washington, DC United States

**Keywords:** artificial intelligence, breast neoplasms, survivors, insomnia, cognitive behavioral therapy, mobile phones

## Abstract

**Background:**

Breast cancer survivors (BCSs) are a growing population with a higher prevalence of insomnia than women of the same age without a history of cancer. Cognitive behavioral therapy for insomnia (CBT-I) has been shown to be effective in this population, but it is not widely available to those who need it.

**Objective:**

This study aimed to better understand BCSs’ experiences with insomnia and to explore the feasibility and acceptability of delivering CBT-I using a virtual assistant (Amazon Alexa).

**Methods:**

We first conducted a formative phase with 2 focus groups and 3 in-depth interviews to understand BCSs’ perceptions of insomnia as well as their interest in and comfort with using a virtual assistant to learn about CBT-I. We then developed a prototype incorporating participant preferences and CBT-I components and demonstrated it in group and individual settings to BCSs to evaluate acceptability, interest, perceived feasibility, educational potential, and usability of the prototype. We also collected open-ended feedback on the content and used frequencies to describe the quantitative data.

**Results:**

We recruited 11 BCSs with insomnia in the formative phase and 14 BCSs in the prototype demonstration. In formative work, anxiety, fear, and hot flashes were identified as causes of insomnia. After prototype demonstration, nearly 79% (11/14) of participants reported an interest in and perceived feasibility of using the virtual assistant to record sleep patterns. Approximately two-thirds of the participants thought lifestyle modification (9/14, 64%) and sleep restriction (9/14, 64%) would be feasible and were interested in this feature of the program (10/14, 71% and 9/14, 64%, respectively). Relaxation exercises were rated as interesting and feasible using the virtual assistant by 71% (10/14) of the participants. Usability was rated as better than average, and all women reported that they would recommend the program to friends and family.

**Conclusions:**

This virtual assistant prototype delivering CBT-I components by using a smart speaker was rated as feasible and acceptable, suggesting that this prototype should be fully developed and tested for efficacy in the BCS population. If efficacy is shown in this population, the prototype should also be adapted for other high-risk populations.

## Introduction

### Background

There were an estimated 3.6 million breast cancer survivors (BCSs) in the United States in 2016, and an estimated 30% to 50% of BCSs suffer from insomnia [1-3]. Insomnia, the most prevalent sleep disorder, has detrimental health consequences on cardiometabolic and immune system health, neurobehavioral function, depression, fatigue, and quality of life [4-7]. Poor sleep efficiency, duration, and quality have also been associated with an increased risk of mortality in BCSs [8,9]. Causes of insomnia may be multifaceted, including cancer-related physiological processes, iatrogenic effects of oncotherapies, menopause, and comorbid mood disorders associated with cancer diagnosis and psychosocial and economic stressors [10,11]. Insomnia after breast cancer treatment is often persistent, lasting multiple years, but is not frequently discussed with cancer care providers [12].

Cognitive behavioral therapy for insomnia (CBT-I) is recommended as the first-line treatment by the American College of Physicians and the National Comprehensive Cancer Network and has shown efficacy in a BCS population [13-15]. However, CBT-I–trained practitioners are scarce, it may not be covered by insurance, and scheduling multiple follow-up visits for insomnia can inhibit completion due to competing demands on time and finances [16]. Although automated therapies delivered via the internet have been developed and shown to be effective [17], there is still a need to reach more patients, underscoring the need to create more user-friendly experiences [18]. As screening and treatment for breast cancer improves and survivors live even longer, this growing population will increasingly need feasible options for accessible insomnia treatment [3].

### Objectives

The aim of our study was to better understand BCSs’ experiences with insomnia and to inform the development of a prototype through focus groups and in-depth interviews that explore how BCSs would perceive and use a screen-free, voice-activated program for CBT-I. We created a series of *metrics of success*, which we set out to meet before moving forward with fully developing the prototype and conducting efficacy testing. In this formative work, we solicited responses from survivors distinct from those who gave formative feedback. Our goals were to achieve that the majority of participants would report that they were *somewhat* to *very* interested in using the technology, that the majority of participants would report increased knowledge of CBT-I, and that participants would rate the concepts presented as *somewhat* to *very* important in addressing insomnia. We also set a target that a majority of participants would rate the perceived feasibility of using this prototype for the delivery of CBT-I components as moderate to high and that the prototype would show better than average system usability (score >68).

## Methods

### Study Participants

We recruited women through the George Washington University Medical Faculty Associates (GW MFA) in conjunction with the breast cancer team. We also advertised in the local newspaper, reached out to local breast cancer survivorship groups, and mailed flyers to GW MFA patients who met the basic eligibility criteria. BCSs were considered eligible if they had a history of stage I-III breast cancer and had completed active treatment (ie, surgery, radiation, and chemotherapy) at least three months prior. We used the Pittsburg sleep quality index, which has been validated in this population [19], to screen women for insomnia symptoms and severity. We used a previously established threshold of a score ≥5 (out of 21) to identify women eligible for participation [20]. In total, we reached out to 63 women; 25 women did not respond to contact, 2 women were stage 4 and thus not eligible, 3 women said they were not interested, 4 women were confirmed to participate but did not show up in focus groups, and 4 women had scheduling conflicts with the proposed dates. We sequentially enrolled women first in the formative, qualitative phase of the study until our target was reached and then in the prototype demonstration. In step 1 (formative), 11 women were included, and in step 2 (prototype demonstration), 14 women were included.

### Formative Work

All formative data were gathered using focus groups in person or using in-depth interviews in person or by phone. The focus groups and in-depth interview guide questions are outlined in Table 1. These guides were semistructured, and facilitators were encouraged to probe participant responses. We were interested in perceptions of insomnia and how it might relate to breast cancer diagnosis, perceived triggers and symptoms of insomnia, anticipated barriers to CBT-I adherence, and comfort with using smart speaker technology. We, thus, inquired about participant experiences with insomnia, including the type of symptoms, timing of onset, and attempts to treat insomnia. We also asked women about their comfort level by using virtual assistants such as smart speakers like the Amazon Alexa or Google Home and smartphone-based assistants like Apple’s Siri on the iPhone. Our questions were open-ended, with probes to better understand the current knowledge of both insomnia treatment options and smart speakers.

We conducted 2 focus groups. In group 1, 6 women participated. In group 2, only 2 women attended out of the 7 scheduled to attend. Last-minute conflicts came up with childcare (n=1), family emergencies (n=3), and rescheduling requests (n=1). Thus, we scheduled in-depth interviews with additional participants. We stopped focus groups and in-depth interviews when we found that responses did not generate any new information beyond what we had already collected.

Media Rez LLC, a Washington DC–based technology company, developed the prototype, which we called *Sleep Helper*. Media Rez drew on multiple sources for developing the Sleep Helper program, using the standardized CBT-I protocol that has been evaluated in extensive scientific literature and has demonstrated efficacy in similar populations [15], input from research team members with experience in delivering CBT-I, and target population input. All coding was performed by HA and reviewed by the study team. Researchers used emergent themes from formative data to further frame content and create user appeal. The prototype included the following modules, based on CBT-I components: *morning*, consisting of recording time in bed and time sleeping, number of awakenings, and length of awakenings; *evening*, consisting of recording caffeine and alcohol intake, exercise, napping, and the ability to set reminders for the morning so as to help clear the mind; *education*, with short guidance about sleep hygiene; and *relaxation*, including an example meditation music and script. The scripts are shown in Table 2.

Some scripts are dependent on the participant’s unique responses and only play when the participant provides a particular answer. For example, scripts might include suggestions for improved sleep hygiene based on the participants’ answers. Extensive scripts allow a wide variety of answers, so they feel more natural and identify nonresponsive or unclear answers to be able to direct the participant back to the question.

**Table 1:** Focus group or interview discussion questions.

Theme	Questions
1. Perceptions and experiences regarding insomnia	When did your insomnia start, and how would you describe it to others? How has insomnia affected your cancer survivorship experience?
2. Perceived triggers and symptoms of insomnia	Can you tell me about a specific thing that triggers your insomnia? What strategies have you tried to overcome your insomnia? What kinds of things do you do when you can’t sleep?
3. Anticipated barriers to cognitive behavioral therapy for insomnia	What are your thoughts on changing lifestyle, diet, and other habits to improve your sleeping patterns?
4. Current usage of Amazon’s Echo/Dot, Google Home, or similar devices	Do you use a home device that uses artificial intelligence at present, such as an Amazon Echo or Google Home? This can also include Apple’s Siri or other mobile devices. If you have ever seen or used one, can you describe that experience?
5. Comfort in interacting with voice-activated AI^a^ assistants	Tell me how you would feel about using voice technology at home to work on strategies to improve insomnia symptoms? Have you used AI or other equivalent technology (cell phone or computer applications) for health or mood-related issues? What were the strengths (frequency, reminders, interactive, etc) of using this technology? What were the weaknesses?
6. General thoughts on using the smart speaker (Alexa) to address insomnia	What are your hopes for insomnia treatment? What frustrations do you foresee in using a device rather than speaking to a human being? What are your thoughts on having Alexa control lighting and temperature in your bedroom? How interested would you be in using a smart speaker to: learn about the stages of sleep, strategies to overcome insomnia, keep a sleep diary, connect with a specialist, or hear relaxation exercises? How concerned would you be about using a smart speaker in regard to privacy? How important are issues such as personalization and the ability for the smart speaker to meet your needs and answer questions?

^a^AI: artificial intelligence.

**Table 2:** Example scripts for Sleep Helper modules.

Module name		Cognitive behavioral therapy for insomnia component
**Education**		
	Congratulations! You left the bed three times this week when you couldn’t sleep, and listened to an audiobook until you were tired enough to sleep. This is good, because it helps you associate the bed only with sleep. In this case, sleep restriction could be helpful. Instead of going to bed at 10 pm, would you like to try going to bed at 10:30 for the next few nights?	Psychoeducation and stimulus control
	(if yes) Good. I’ll reset your alarms and lights for 10:30, and we’ll see how that works out.	Sleep restriction
**Morning**		
	I would like to ask you how easily you fell asleep last night, on a five-point scale, where one means it was very difficult to fall asleep, and five means you fell asleep easily.	Sleep hygiene
	I’m curious to know how refreshed you feel now, on a scale from one, meaning fatigued, to five, meaning refreshed.	Sleep hygiene
	How many times did you awaken last night?	Sleep hygiene
**Night**		
	Sometimes it’s hard to sleep because of unfinished business on our minds. I can help by remembering anything that’s on your mind, and reminding you of it in the morning. Is there something you would like me to remember for you?	Psychoeducation and relaxation (reduce running thoughts or anxiety)
	Did you exercise at least once today for more than 20 minutes?	Sleep hygiene
	How many caffeinated drinks did you have between twelve o’clock noon, and bed time?	Sleep hygiene
	Some people find that avoiding caffeine after twelve o’clock noon makes it easier to sleep at night. Would you be interested in trying to avoid caffeine after noon?	Psychoeducation
	(if yes) Great! Going forward, I’ll make recommendations to cut back on afternoon caffeine, and track your progress.	Sleep hygiene
**Relaxation**		
	The last thing I can do for you tonight is to begin a relaxing meditation sequence. Would you like to begin this relaxation?	Relaxation exercises

### Prototype Testing

After developing the prototype, we demonstrated the *Sleep Helper* program to 14 BCSs who had not shared formative input to measure interest, feasibility, and knowledge of the key components of CBT-I and smart speaker features. Demonstrations ranged from 60 to 90 min and included researcher prompts, observing participants engaging with the prototype, and soliciting feedback. Our objective was to determine the acceptability and teaching potential of the virtual assistant in delivering key CBT-I skills to BCSs. We completed 3 group demonstrations of the prototype (n=3, n=3, and n=6 participants) and additional individual presentations (n=2) to accommodate scheduling preferences. Our primary outcomes of interest, feasibility, and perceived importance were measured using a 5-point Likert scale at the end of the demonstration. We also measured usability of the prototype using the system usability scale (SUS), a 10-item scale with a 5-point Likert scale, with options ranging from strongly disagree to strongly agree for each item. Example items include *I would imagine that most people would learn to use this system very quickly* or *I thought that the system was easy to use*. The SUS is easy to administer, can be used in small sample sizes, and has shown validity in differentiating usable from unusable systems [21]. Previous research suggests that scores above 68 indicate better than average usability [22]. Finally, participants completed a written survey on whether their knowledge of CBT-I had improved from pre- to postdemonstration and whether they would recommend the prototype to friends or family, based on what they had seen.

This study was approved by the George Washington University’s Institutional Review Board. All participants read the informed consent forms and agreed to participate before initiating focus groups or interviews.

## Results

### Participants

Our 25 participants were aged, on average, 58.5 (SD 9.8) years; 72% (18/25) of participants reported a history of stage I breast cancer, 24% (6/25) reported stage II breast cancer, and 4% (1/25) reported stage III breast cancer. Women reported completing curative treatment (surgery, radiation, and chemotherapy), on average, 57.1 months ago (SD 60.5 months). A total of 12 participants were self-reported as black or African American, 11 as non-Hispanic white, and 2 preferred not to answer.

### Formative Work

For exploratory, open-ended qualitative interviews and focus groups, we recruited 11 BCSs with insomnia to describe their interest in and perceived feasibility of using voice-activated smart home technology for delivery of CBT-I components.

Open-ended questions about experiences with insomnia outlined the perceived causes and symptoms experienced by participants. Although the majority of participants had previously considered the relationship between their symptoms of insomnia and cancer diagnosis, 2 participants had not previously considered that insomnia might be related to breast cancer until they learned that other survivors in the focus group had similar experiences. Both focus groups reported similar experiences with insomnia, including a *new normal* of deficient sleep and low energy after either having trouble falling asleep or staying asleep (ie, being awake in the middle of the night). Common perceived triggers and symptoms of insomnia included anxiety, hot flashes, continuous need to go to the bathroom, and disruptive thoughts occurring during the intended sleep period. One woman described her trigger as:

Clearly anxiety. I’m just very, very anxious about something. Physically, something doesn’t feel right, it’s hard to sleep because it’s on your mind.

Other women attributed sleep disturbances to physical disruptions from hormonal therapies, for example:

It was practically the first night on tamoxifen, I was up every 2-3 hours having to go to the bathroom or having hot flashes. And this has been going [on] for 2 years. So, I have pretty much had 2 years of not having much sleep.

The 2 most commonly mentioned strategies used to overcome insomnia included using technology such as a cell phone or television to distract themselves from anxious thoughts and keeping the room temperature cool and comfortable. Some women were familiar with strategies of restricting liquids before bedtime, avoiding alcohol and caffeine, and limiting naps, but participants also expressed difficulty in changing these behaviors.

Participants were eager to have customizable features to be able to personalize the program, but they also expressed a need for simplicity and straightforward use. As one woman expressed “one size does not fit all, you have to acknowledge that what works for one person may not work for someone else*.*” Other women found the device “really attractive [in] that it’s all put together in [one] package” but also “needs to offer, a kind of intuitive straightforward easy to use process.”

Women largely had some, but not extensive, experience with using smart speakers. Most participants were not concerned about the security of sharing information about sleep with a smart home device, but they did want information about how data were going to be used. Other issues such as frustration using the device (based on prior voice-activated programs such as Siri not understanding commands) and concerns “if [the smart speaker] started talking randomly” were mentioned. Women also noted that they would not want it to disturb a bed partner (eg, having the device talk out loud at night).

### Prototype Testing

We enrolled 14 women who received the prototype demonstration. We demonstrated the *morning*, *evening*, *education*, and *relaxation* modules, asking participants open-ended questions about what they thought of the content and how they might or might not want to incorporate it into their home routines. Participants responded that the *morning* and *evening* modules were of an appropriate length and that features such as querying on caffeine were good reminders about changing behaviors. “I’ve been trying to do that [note time of last caffeine intake] on my own but haven’t been entirely successful.” Some women suggested wanting encouragement and being congratulated for meeting goals (eg, if they went to sleep at the recommended time for a few days in a row). Participants found that the educational module was *surprisingly engaging*, but suggested that they might want a *menu* of choices to know what kind of things they might be able to listen to; this feature will be available on the app that accompanies the virtual assistant program. Most of the participants were happy with short lessons but liked the idea that they could ask the Sleep Helper program to tell them more about a given subject. Participants expressed concerns over privacy but thought that they would probably use the program anyway, saying that they share their data already with other programs and that as long as they knew how data were being used, they would be reassured. Others thought that the benefit of addressing sleep concerns outweighs the risk:

Because it is for a specific purpose...it is not to make my life easier it is to give me knowledge, data, and help me rather than just for entertainment purposes...

When asked about how long they could imagine using the program for, some women stated that:

After 30 days everything becomes a routine...you would look forward to going in there and talking to it.

This suggested that they envisioned continuing to use the program on an ongoing basis to record patterns even if sleep had improved. Others were skeptical about wanting to report sleep patterns daily but liked the idea that they could create default settings and then only update things that changed that day. Women also suggested allowing for customization for individual life events or vacations that may affect sleep patterns. “It should ask did something [that affected your sleep] happen today?” When women were asked how much guidance they needed to use the device, they said that a voice-activated setup guide would be sufficient, although a few women thought they might want to have a number to call if they needed support in using the program.

After the demonstration of the key prototype features, and open-ended discussion, participants completed questionnaires. On postdemonstration questionnaires, 79% (11/14) of the BCSs reported that knowledge of insomnia and CBT-I had increased after using the prototype compared with when they had arrived; the remaining participants said they had about the same knowledge after the demonstration. All 14 participants confirmed interest in using the program to treat insomnia symptoms at home, and all the participants reported that based on the demonstration, they would recommend the program to friends or family.

All participants completed Likert scale questionnaires about interest, perceived feasibility, and importance of key CBT-I concepts that we had built into the initial prototype (Table 3). In short, nearly 79% (11/14) of participants thought it was both of interest and would be feasible to use the Sleep Helper program to record sleep patterns. Nearly two-thirds of the participants thought it would be feasible to tackle the difficult challenges of lifestyle modification (9/14, 64%) and sleep restriction (9/14, 64%) and were interested in this feature on the Sleep Helper program (10/14, 71% and 9/14, 64%, respectively). Using relaxation exercises on the Amazon Alexa were cited as of interest and feasible by 71% (10/14) of participants. More participants indicated that they had neutral feelings about the importance of using the bed only for sleep and sex, leaving the bed after 20 min of awake time, and keeping a regular schedule for getting in and out of bed, indicating potential educational opportunities to increase perceived salience.

Importantly, all participants said that they would recommend the prototype to friends or family, showing strong potential for future testing. The average SUS score was 82.3 (range 50-100), indicating success in meeting the target usability score of ≥68.

**Table 3:** Interest, perceived feasibility, and importance of prototype (N=14).

Interest, feasibility, and importance		Five-point Likert scale response, n (%)				
		Very	Somewhat	Neutral	Not very	Not at all
**How interested would you be in using the Amazon Alexa to...**						
	Record your daily sleep pattern	11 (79)	3 (21)	0 (0)	0 (0)	0 (0)
	Prompt you to change behaviors such as maintaining a regular schedule, avoiding stimulants, exercising, and avoiding screen time at night	10 (71)	2 (14)	2 (14)	0 (0)	0 (0)
	Practice guided relaxation	10 (71)	3 (21)	0 (0)	1 (7)	0 (0)
	Deliver a visual prompt to let you know that 20 min are up and you should leave the bed	9 (64)	2 (14)	1 (7)	1 (7)	1 (7)
	Prompt you to restrict time spent in bed	9 (64)	2 (14)	1 (7)	0 (0)	1 (7)
**How feasible do you think that it would be to...**						
	Tell Alexa when you went to sleep and nighttime awakenings when you wake up in the morning	11 (79)	2 (14)	0 (0)	0 (0)	1 (7)
	Change behaviors such as having a regular schedule, avoiding stimulants, exercising, and avoiding screen time at night	9 (64)	5 (29)	1 (7)	0 (0)	0 (0)
	Practice relaxation exercises to help your insomnia symptoms	10 (71)	3 (21)	1 (7)	0 (0)	0 (0)
	Leave the bedroom if you do not fall asleep within 20 min	10 (71)	1 (7)	1 (7)	2 (14)	0 (0)
	Restrict the amount of time you spend in bed	9 (64)	3 (21)	1 (7)	0 (0)	0 (0)
**How important do you think that...is in avoiding insomnia**						
	Understanding the role of sleep in health	13 (93)	0 (0)	1 (7)	0 (0)	0 (0)
	Behaviors such as having a regular schedule, avoiding stimulants, and avoiding exposure to screens at night	12 (86)	1 (7)	1 (7)	0 (0)	0 (0)
	Relaxation exercise	12 (86)	2 (14)	0 (0)	0 (0)	0 (0)
	Using the bed only for sleep and sex and leaving the bed if you cannot sleep for 20 min	7 (50)	3 (21)	3 (21)	1 (7)	0 (0)
	Keeping a regular schedule for getting in and out of bed	8 (57)	3 (21)	3 (21)	0 (0)	0 (0)

## Discussion

### Principal Findings

In our formative work to understand BCSs’ experiences with insomnia and smart home devices, we found that participants were interested in this technology, particularly if it could be personalized for ease of use. We reached our goal that the majority of participants would report that they were *somewhat* to *very* interested in using the technology, that participants would rate the program as feasible and highly usable, and that the majority of participants would report increased knowledge of CBT-I. In our demonstration, participants also reported that sleep logs (one of the key components of CBT-I that clinicians depend on to proscribe sleep recommendations) using the prototype was very feasible. This suggests that the data collected by the smart home device can be used by artificial intelligence programming to create personalized recommendations and schedules that go beyond simply presenting sleep hygiene education.

### Comparison With Prior Work

Another qualitative study of cancer survivors similarly showed that insomnia may be exacerbated by anxiety, inability to relax, and use of screen time before bed [23]. Each of these issues also surfaced in our interviews and focus groups and were considered in prototype development.

Previous studies have suggested a need for scalable methods to deliver CBT services, with self-administered CBT-I as a first step in a stepped care model [24]. Our study is not the first to use technology to offer components of CBT-I to cancer survivors. Researchers have previously delivered automated CBT-I to BCSs (n=255) via a web-based portal, showing improvements in sleep outcomes for wake after sleep onset and insomnia severity in a randomized controlled trial [25]. Another study among 18 BCSs and 10 other cancer survivors on average 4 years after diagnosis demonstrated the efficacy of web-based CBT-I on the overall insomnia severity index as well as using sleep diary measures [26]. A larger study of the same program including 303 adults with chronic insomnia (not specifically cancer survivors) supported the efficacy of this intervention in improving sleep outcomes [27]. These results suggest the promise of using an automated, technology-driven portal to deliver components of CBT-I to cancer survivors. Still, our approach of using voice-activated smart home technology differs from web- or video-based technologies, as it may have increased reach (eg, relaxation scripts could be delivered in the bed at the point of going to sleep using facemasks with built-in speakers), may have increased frequency of contact, and may eliminate screen time, which is one of the triggers for sleep disruption.

### Strengths

The strengths of our study include formative work, development of an innovative technology, and early user testing to offer feedback on areas for improvement as the product is further developed. We triangulated data from the scientific literature and from formative data collection to frame messages around insomnia that were specific to our target population of BCSs.

### Limitations

In this formative work, we gathered information from a limited number of participants, partly due to the short time frame of study funding. Yet, by the end of data collection, we were not observing additional themes that emerged, suggesting feedback saturation. We also demonstrated only a limited prototype, as the device was not fully programmed to incorporate feedback or be used in home testing. However, as our objective during this phase was only to assess perceived feasibility, this should not be considered a major limitation at this point in time. In future studies, we plan to further develop and test this prototype for actual feasibility and efficacy. Furthermore, we did not have demographic information such as income or education, which may have affected participant responses.

### Conclusions

We anticipate that by using an iterative development process with end users to ensure high user satisfaction, we will be able to further develop a voice-activated program to deliver CBT-I components that will improve insomnia among our target population of BCSs. In the long term, we hope to increase the uptake of this effective therapy in the breast cancer population beyond what has been achieved with in-person visits, videos, or website-based programs. After demonstrating efficacy in the BCS population, we plan to adapt the technology for other high-risk populations.

## Abbreviations

BCSs: breast cancer survivors
CBT-I: cognitive behavioral therapy for insomnia
GW MFA: George Washington University Medical Faculty Associates
SUS: system usability scale
